# Feasibility and acceptability of experience sampling among LGBTQ+ young people with self-harmful thoughts and behaviours

**DOI:** 10.3389/fpsyt.2022.916164

**Published:** 2022-08-17

**Authors:** A. J. Williams, Jon Arcelus, Ellen Townsend, Maria Michail

**Affiliations:** ^1^Institute for Mental Health, School of Psychology, University of Birmingham, Birmingham, United Kingdom; ^2^Self-Harm Research Group, School of Psychology, University of Nottingham, Nottingham, United Kingdom; ^3^Informatics, Faculty of Natural, Mathematical and Engineering Sciences, King’s College London, London, United Kingdom; ^4^Institute of Mental Health, School of Medicine, University of Nottingham, Nottingham, United Kingdom; ^5^Bellvitge Biomedical Research Institute (IDIBELL), University of Barcelona, Barcelona, Spain

**Keywords:** self-harm, experience sampling method (ESM), LGBTQ+, young people, feasibility, acceptability

## Abstract

This study was the first to determine whether it was feasible and acceptable to use experience sampling methods (ESM) among LGBTQ+ young people, who had current experiences of self-harm. Sixteen LGBTQ+ young people (16–25 years old) took part in the experience sampling study. This included a baseline assessment, a 7-day ESM assessment (participants were sampled six times a day using a phone app), and the option of an interview at the end of the 7-day ESM assessment. Feasibility data was descriptively analysed, with pilot ESM data presented. Qualitative data was thematically analysed to determine the acceptability (barriers and facilitators) of taking part in this study. Study feasibility was assessed by enrolment rate (55.2%), participant retention across assessment period (100%), ESM app feasibility (87.5%), and good adherence to total number of ESM surveys (67.6%). Individual study adherence ranged between 43 and 95.2%. Study acceptability was assessed by participant interviews. Thematic analysis indicated four superordinate themes; (i) Self-reflection and awareness; (ii) Practicalities of ESM surveys; (iii) Daily timeframes; and (iv) Suggestions for future studies. Pilot ESM data demonstrates that there was fluctuation of depressive and anxiety symptoms within- and between- participants over the course of the study, however, greater sample power is needed for full analysis. This study demonstrated that ESM designs are feasible and acceptable among LGBTQ+ young people with current experiences of self-harm. Pilot data indicated that specific experiences and moods are likely to be important to self-harm. These potentially have a temporal influence on self-harm behaviour or ideation, and therefore should be examined in a fully powered sample.

## Introduction

Self-harm (the injury or poisoning of self, irrespective of suicidal intentions) (1) is a significant issue among young people. Globally, self-harm, with suicidal intentions, is the fourth leading cause of death for those 15–19-years (2). Among young people in the United Kingdom, it is estimated that between 13.2 and 19.7% struggle with self-harm (3–5). Among studies which focus solely on LGBTQ+ young people, self-harm prevalence was found to be between 8 and 33% (6, 7), which is higher on average than prevalence among cisgender, heterosexual counterparts (3).

LGBTQ+ young people face uniquely stressful experiences relating to their sexual orientation and/or gender identity (8–10). Experiences such as internalised self-hatred, negative responses from family, and bullying or victimisation are key to self-harm (11, 12). However, less is known about how such experiences may be time-variant (the close interaction between the event and the behaviour). Investigating how stressors may influence self-harm across hours or days, rather than weeks and years, would aid self-harm prevention (13). For example, in their study, Lockwood et al. (14) found that young people often reported that when self-harm occurred, it was within ten minutes of having experienced a self-harm thought (though not all thoughts led to self-harm enaction). This indicates not only that impulsivity was a predictor of self-harm (14) but also that precipitating experiences may have a time-variant influence on self-harm thoughts or behaviour. To explore real-time influences, experience sampling methods (ESM; also known as Ecological Momentary Assessment, EMA; (15–17)) can be used. ESM offers a temporal understanding of the sequence in which events, experiences, moods or cognitions may occur and how they relate to each other (17).

Experience sampling methods has effectively been used to investigate self-harm fluctuation and experiences which have temporal influence across various populations (18–26). While ESM has been used among highly vulnerable populations including those with eating disorders, psychosis, borderline personality disorder, and depression (19, 23–25, 27), often these studies focus on participants who are 18-years old and above. Two previous studies were specifically conducted to determine the feasibility of using ESM with adolescents who engaged with self-harm with suicidal intentions (28, 29). Both studies offered insight that daily assessment of self-harm with suicidal intention was feasible with young people, however one was set within acute psychiatric care (29) and the other following discharge (28). These studies demonstrate that among highly vulnerable young people, ESM is still considered acceptable and feasible to use. However, these were based within clinical services, therefore it there is little information regarding the feasibility of ESM within young people in community settings. Additionally, there is very limited research which considers LGBTQ+ individuals and self-harm.

Fehling (19) assessed 21 sexual orientation minority adults using the LifeData app-system over a period of 2 weeks, to examine the fluctuations of minority stress, Non-Suicidal Self-Injury (NSSI) and mental health difficulties. The study found that greater experiences of minority stress were related to high predictions of distress and engagement with NSSI. Increased rates of NSSI took place at the same timepoints as minority stress events, which indicates a strong temporal relationship between these events and the NSSI behaviour (19). In their studies, Livingston et al. (30, 31) also evaluated the impact of minority stress, in the form of microaggressions, to determine their contribution to psychological distress and substance use within 50 LGBTQ+ adults. These experiences were assessed over two weeks using Basic for Android, which was installed onto Samsung Galaxy phones. This study indicated that high psychological distress and maladaptive coping behaviours (e.g., substance use) were predicted by experiencing microaggression 2–3 h previously. While this pool of literature is small, it evidences that minority stress experiences can have real-time impact on mood, distress, and self-harm. However, Livingston et al. (30, 31) did not explore self-harm, and Fehling (19) only considered NSSI in LGB adults within their sample.

This highlights a clear gap in the ESM literature considering the experiences of LGBTQ+ young people with current experiences of self-harm. This study would be the first to determine whether it is feasible and acceptable to conduct an experience sampling study with LGBTQ+ young people, who have current experiences of self-harm, with and without suicidal intentions. Specific objectives are listed;

• To determine feasibility; recruitment and consent rates, retention, app usability and adherence will be examined.
• To assess acceptability; LGBTQ+ young people’s views of the barriers and facilitators to engaging with the ESM study will be explored.
• Study parameters are considered using pilot ESM data, to indicate whether a follow-up study would be worthwhile. Firstly, using the study design, sample size will be determined through a power calculation. Secondly, pilot ESM data will be observed to examine whether there is any fluctuation of ESM items within- and between- participants.

## Materials and methods

### Participants

Participants were recruited using online social media platforms and MQ’s mental health research website; Participate^1^ between 14th, June 2021 and 24th, August 2021. To take part, participants had to meet five inclusion criteria: (i) identify as any part of the LGBTQ+ umbrella; (ii) currently experience self-harmful thoughts and/or behaviours, with or without suicidal intentions; (iii) be aged between 16 and 25 years old; (iv) be registered with a United Kingdom based GP practice; and (v) have personal access to a smartphone.

Participants received a £10 voucher as compensation for completing the full-study (phase 2 + phase 3) or £5 if they completed either the full ESM period (phase 2-only) or withdrew during the ESM period but took part in the semi-structured interview (phase 3-only).

### Measures and procedures

This is a mixed-method experimental study which uses ESM over a 7-day period (six prompts per day between 8:00 and 22:00) with LGBTQ+ young people who have experiences of self-harm, with and without suicidal intention. The design was informed by the LGBTQ+ advisory group, individuals with lived self-harm experience who offered insights and feedback for the study. This group represents a range of sexual orientations and gender identities. Ethical approval was received from the Science, Technology, Engineering and Mathematic Ethical Review Committee on the 8th of June 2021 (ERN_201745). The study was pre-registered on the Open Science Framework following the ESM template developed by Kirtley et al. (32), study pre-registration: DOI 10.17605/OSF.IO/DPWT.

**FIGURE 1:**
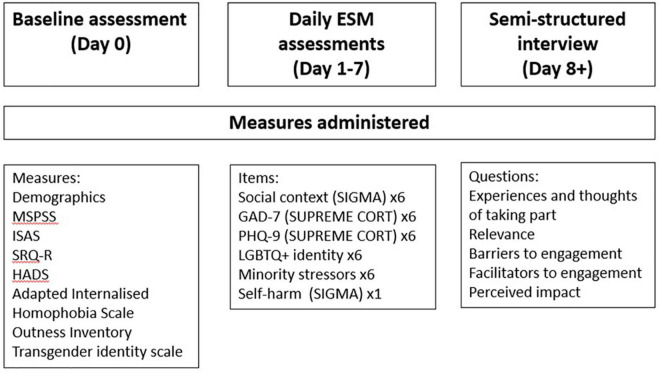
Overview of data collection phases.

The study includes briefing and debriefing, while data collection took place over three testing phases: (i) baseline assessment (Phase 1); (ii) 7-day ESM assessment (Phase 2); and (iii) post-ESM semi-structured interview (Phase 3). An overview of these phases can be seen in Figure 1. Phases one and two were designed to test the feasibility of conducting an ESM study with this population, and therefore follow the traditional structure of ESM studies (24, 29). Phase three explored participants’ own perceptions and experiences to determine how acceptable the study was, as well as discuss facilitators or barriers to engagement with ESM.

#### Phase 1: Baseline assessment

Phase one of the study was to complete an online baseline assessment which was hosted by Qualtrics. The link was sent to participants once their completed, signed consent form had been received and checked. The baseline assessment took between 20 and 30 min to complete. This was to determine whether participants would complete a stand-alone questionnaire followed by the 7-day ESM assessment. For a follow-up study, this would be used as cross-sectional snapshot of participant profiles and considered in relation to their prospective data.

Demographics which confirmed the study inclusion criteria were collected: age, country, sexual orientation, and gender identity, as well as ethnicity and occupation. Following this, participants completed a series of validated measures, which have all previously been used with LGBTQ+ populations with good internal consistency. All participants completed the first 6 measures, before the 7th measure, a binary, branching question was included. If participants were transgender or gender diverse (TGD) they would complete the final two measures. On completion of baseline assessment, the data was checked to ensure that participants met the inclusion criteria before conducting briefing.

##### Multidimensional scale of perceived social support

A 12-item scale used to measure the perceived social support from family, friends, and significant others (33). A 7-point Likert scale is used, from 1 (very strongly agree) to 7 (very strongly disagree). The measure includes three subscales which each focus on a type of support (family, friends, significant others). Cronbach’s alpha of the Multidimensional scale of perceived social support (MSPSS) was excellent (α = 0.89) with subscales ranging between 0.93 and 0.96. Mean total and subscale scores range from 1 to 7, with higher scores suggesting greater perceived social support.

##### Inventory of statements about self-Injury (ISAS)

There are two sections to the ISAS (34, 35); (i) assessment of lifetime frequency of 12 self-harm behaviours; and (ii) assessment of 13 functions of self-harm over 39 questions, which relate to either intra- or interpersonal functions. Each of these questions is rated on a 3-point Likert scale from 0 (not relevant) to 2 (very relevant). Scores for each self-harm function range from 0 to 6. Strong internal consistency was achieved for intrapersonal functions (α = 0.84), while interpersonal functions consistency being 0.65.

##### Suicidal behaviours questionnaire-revised (SRQ-R)

A 4-item scale to determine suicide risk from thoughts, behaviours, frequency, and intention (36). Items use either a 5- or 7-point response scale. Total scores range from 3 to 18, with higher scores suggesting greater suicide risk. Consistency was indicated Cronbach’s alpha (α = 0.44).

##### Hospital anxiety and depression scale (HADS)

This 14-item scale is used to indicate anxiety and depressive symptoms (37). Subscales are calculated to indicate presence of depression (α = 0.58) and anxiety (α = 0.78) separately, using a 4-point Likert scale. Individually, scores from 0 to 7 are considered normal (no symptoms), 8 to 10 suggesting possible symptoms, and scores of 11 or higher indicating likelihood of a disorder.

##### Adapted internalised homophobia scale

This measure was used to indicate negative self-perception in relation to LGBTQ+ identity, across five questions (31, 38). A 5-point Likert scale was used, from 1 (strongly disagree) to 5 (strongly agree), with higher scores indicated more negative associations with LGBTQ+ identity. Cronbach’s alpha was 0.78.

##### Outness inventory

This measure was used to assess the level of which participants were “out” about their LGBTQ+ identity across 13 groups of social relationships, ranging from mother to leaders of religious community to peers (31, 39). All items were assessed using a 7-point Likert scale, from 1 (person definitely does not know about your sexual orientation or gender identity) to 7 (person definitely knows about your sexual orientation or gender identity, and it is openly talked about). Lower scores indicate greater concealment of LGBTQ+ identity. Cronbach’s alpha was 0.74.

##### Transgender identity survey

Twenty-six-items assessed how TGD young people felt about being gender diverse over the last 3 months (40). All items were rated between 1 (strongly disagree) and 7 (strongly agree). The measure consists of four subscales considering pride, passing, alienation, and shame. For the total score, the pride subscale is reverse scored, such that a higher score indicates greater internalised transphobia. For the full measure, Cronbach’s alpha was 0.96 with subscales ranging between 0.85 and 0.95.

##### Congruence and life satisfaction scale (GCLS)

The overall aim of the GCLS is to measure changes in gender congruence, body satisfaction, mental health and life satisfaction for TGD people (41). This measure contains 7 subscales relating to genitalia, chest, other secondary sex characteristics, social gender role recognition, intimacy, psychological functioning, and life satisfaction. These cluster into two subgroups i) gender congruence (α = 0.90) and gender-related mental well-being and general life satisfaction (α = 0.93). These are rated on a 5-point Likert scale; 1 (always) to 5 (never), with higher scores indicating positive outcomes.

The Zoom ESM briefing was arranged at participants’ convenience. It was mandatory for participants to attend this briefing, however, having their camera on was optional. During the briefing, participants were introduced to the study and explained the study procedures. Participants were asked to download the mEMA app which hosted the 7-day ESM assessment. The first author would ensure that they were able to log onto the app using their confidential mEMA code and had access to their ESM surveys. A dummy run of a “prompt” was conducted (push notification on a smartphone). During this dummy run, participants were led through the different types of questions and explained the rating scales. Participants were asked if they had any questions about the study overall or the practical aspects of the app.

Following this, safeguarding procedures, participant rights and compensations were explained. Participants were also told that the first author would be in touch on day 2 of the 7-day ESM assessment to encourage study adherence and troubleshoot any technical issues. Participants were asked to confirm they understood and were happy with all procedures, they were then invited to ask any further questions. The following day, the 7-day ESM assessment would begin.

#### Phase 2: 7-day experience sampling methods assessment

Phase two of the study was the 7-day ESM assessment. This would run for the next consecutive week following participant briefing. The ESM surveys were administered using the mEMA app from ilumivu^2^, software which was designed specifically for ESM research using smartphones. Participants were assigned a confidential code which gave them access to the app, so that no identifying information was shared with the software platform. Survey data was collected and stored on the participants’ smartphones; once an internet connection was established this data would sync with the online platform. This software was designed for multi-platform compatibility, which allows for automated notifications for participants using a quasi-random temporal sampling structure (participants were randomly prompted six times between 8:00 and 22:00). The duration and number of survey notifications followed similar designs to previous research (24, 27, 42). Participants were given a 30-min window to respond to each survey notification, this was to ensure that participants gave in-the-moment responses. The mEMA app was piloted using an Android and an iOS device to ensure its compatibility. The app and online platform received security clearance from University of Birmingham IT security.

##### Experience sampling method items

The ESM items were selected to represent previously identified processes underlying self-harm in LGBTQ+ young people (11, 12). These were grouped thematically; (i) social context and environment (items asking who the participant was with at that time and perceived support); (ii) depression and anxiety; and (iii) perception of LGBTQ+ identity and minority stressors. These items were asked six times a day. The last assessment of each day would also include three items about self-harm and suicidal thoughts, and self-harm behaviour. An overview of all ESM items are presented in Table 1.

**TABLE 1:** Overview of all experience sampling method (ESM) items.

Preceding research	Key finding: risk factor or experience	ESM topic	Origin of item	Number of items	Times asked per day
(11, 12)	Victimisation. Negative responses to being LGBTQ+. Feeling responsible for others.	Social context and environment	SIGMA (44) Two additional items developed and were face validated by LGBTQ+ Advisory Group.	Branching item = 4 or additional branching question. Second item = 7, or 9 further questions.	6
(11)	Mental health difficulties	Depression (PHQ-9) Anxiety (GAD-7)	SUPEREME CORT study (63)	16	6
(12)	Struggling with processing and understanding one’s own LGBTQ+ identity	Perception of LGBTQ+ identity	Items developed and were face validated by LGBTQ+ Advisory Group.	6	6
***Minority stressors***					
(11, 12)	Victimisation. Negative responses to being LGBTQ+.	Discrimination	Items developed and were face validated by LGBTQ+ Advisory Group.	Two items, both which branch to two additional items if response is yes.	6
(12)	Coping with gender dysphoria.	Gender dysphoria	Items developed and were face validated by LGBTQ+ Advisory Group.	1	6
(12)	Negative responses to being LGBTQ+.	Misgendering	Items developed and were face validated by LGBTQ+ Advisory Group.	1	6
***Outcome of interest***					
		Self-harm thoughts	SIGMA (44)	1	1
		Suicidal thoughts	SIGMA (44)	1	1
		Self-harm behaviour	SIGMA (44)	1	1

Experience sampling methods items which had been used in previous research were obtained from www.esmitemrepository.com (43). These items came from two primary sources; SIGMA study (44) and SUPREME CORT (45). All items were presented as consistent visual analogs, using 1–7 Likert scales, this has been suggested to reduce participant error (46). Full information of ESM items and the structure of the ESM survey can be found in Supplementary material 1.

#### Phase 3: Post-experience sampling method interview

On the final day of the 7-day ESM assessment, participants were sent an email thanking them for taking part in the study, reminding them that this was the last day, and inviting them to Phase 3 of the study. Phase 3 was a semi-structured interview arranged at participants’ convenience following the receipt of a completed, signed consent form. Interviews took place over Zoom and were audio-recorded using a Dictaphone. Participants were encouraged to speak openly about their opinions, perceptions, and experiences of the study. The interviews lasted a mean of 19 min (12′ to 41′). Following the interview, participants were thanked, debriefed, and compensated for their time.

### Safeguarding procedures

To ensure the safety of participants, several measures were taken. These were explained to participants prior to providing consent for the study and during the study briefing. On enrolment to the study, letters were sent to the participants’ GP practice. This would explain that the individual was involved in a mental health study at the University of Birmingham and provide the first authors’ contact information. No information was presented that this was a self-harm or LGBTQ+ study to avoid unwanted disclosure for the participant. However, GPs were informed that if the participant was experiencing high distress, their practice would be contacted by letter and phone call.

During the 7-day ESM assessment, if a participant scored suicide ideation highly (scores of 6 or 7) or that they had self-harmed, they would receive a pop-up note that acknowledged their distress and advised contacting their GP service or helplines such as Samaritans. Alongside this, data was monitored once a day to assess for self-harm risk (29). Following previous research, a cut-off score for high-risk responses was established (23, 29). This was scoring highly for suicidal thoughts (scores of 6 or 7) and having self-harmed which would result in a wellbeing call. Data was not checked in real-time; it was established that data checking would occur each morning between 10:00 and 12:00. Any wellbeing calls would take place before 15:00 and were conducted by the first author; this was to ensure that contact with supervisors was conducted during academia hours and GP practices would be open in the event that the call needed to be escalated.

This wellbeing call included encouraging help-seeking to the participant’s GP, assessing the imminent risk of a suicide attempt (plans, timeline, access to means), and conducting a safety planning activity with the participant (47). If the participant was at imminent risk of attempting suicide, they would be informed that confidentiality would be broken to inform supervisors, their GP, and potentially emergency services. This would be an immediate phone call to the GP service, and a formal letter. If it was a weekend and the GP service was closed, the safeguarding procedure automatically reverted to contacting emergency services. However, if participants were not at-risk, no further procedures were taken. At this point, participants would be asked if they wished to continue with the study and reminded that it is their right to withdraw if they so wished. All participants were aware of these procedures and agreed to them when signing the consent form.

### Analysis

All quantitative participant data was analysed in SPSS28. For baseline measures total score and subscales, averages, and standard deviations were calculated to give an insight into the characteristics of the participant sample.

Study feasibility was assessed in four key ways; recruitment rates, retention rates, app feasibility and study adherence. Recruitment rates considered the number of respondents over the recruitment period and final study enrolment rate. Reasons for non-consent were recorded. Secondly, retention was examined across the baseline assessment and 7-day ESM assessment, this was to determine whether a particular phase of the study was less desirable. If participants withdrew during any aspect of the study, they were asked for reasons and invited to the post-ESM interview to discuss their opinions of the study and elaborate on exercising their choice to withdraw. Thirdly, feasibility of the mEMA app was determined by the number of days in which participants were able to log in and give responses. Finally, total study adherence was examined by the number of responses to surveys and descriptives of response patterns. This was followed by adherence breakdown by ESM topic items (e.g., social context, mental health, identity and minority stressors, and self-harm). Participant adherence was assessed through individual study adherence and ESM topic surveys completion. Analysis consists of descriptive statistics.

Study acceptability was assessed using the data from LGBTQ+ young people’s semi-structured interviews. All the interviews were conducted and transcribed verbatim by the first author. Following transcription, all transcripts were imported into NVIVO12 and deductively thematically analysed (48–50) to determine barriers and facilitators of taking part within the study. Line-by-line coding of opinions, perceptions and experiences took place. These were then considered in relation to the research aim, and similarities and differences between codes were collated to develop preliminary subthemes. These were reviewed and discussed between the research team to create the final thematic framework.

Using pilot data, the parameters of the study are considered. This is to inform whether a follow-up study would be worthwhile. Firstly, using the current study design, a sample size calculation was conducted in R. This determines the sample number needed to achieve 80% power to detect an association of medium size (*r* = 0.30) using an alpha of 0.05 (51). This is with the parameters of 42 observations per individual across a 7-day ESM period, and indicates the number of participants needed for multi-level regression models, allowing for analysis of the temporal relationship between ESM items and self-harm. Secondly, total scores for selected ESM items were calculated (anxiety, depression), these were averaged over the day for each participant, offering a daily score of ESM item. The GAD-7 and PHQ-9 had previously been adapted for ESM studies (45). As a note, these ESM items use different scales (1–7) from the originals (0–3) and thus do not offer the validated severity thresholds of anxiety or depression (52, 53). Observation of these ESM items is offered to show within- and between- participant changes in scoring over the 7-day ESM assessment. GAD-7 and PHQ-9 scores are then compared between participants who self-harmed and those who did not.

## Results

The final sample consisted of 16 LGBTQ+ young people, with the average age of 19.2 (SD: 2.7). For full participant details, see Table 2. Twelve participants were cisgender and four were TGD. A total of 37.6% identified as bisexual, whilst other sexual orientations were represented by other participants. One participant did distinguish their bisexuality to also include demisexuality, such that they only feel sexual attraction to someone they have an emotional bond with. Another individual identified as neptunic (attraction to female genders and non-binary individuals). Most participants described themselves as white or white British, and nearly half of the sample were sixth form (age range: 16–18) or college students (43.8%).

**TABLE 2:** Full participant sample characteristics.

Participant	Age (years)	Ethnicity	Occupation	Sexuality	Gender
1	19	White British	University student	Bisexual/demisexual	Cisgender woman
2	24	White	Flexible working hours	Gay	Cisgender man
3	25	White	Currently unemployed	Pansexual	Non-binary
4	22	Asian Malaysian	University student	Bisexual	Cisgender woman
5	18	White	Volunteering	Neptunic	Non-binary
6	19	White	Sixth form or college student	Bisexual	Cisgender woman
7	17	White British	Sixth form or college student	Bisexual	Cisgender woman
8	16	White British	Sixth form or college student	Bisexual	Cisgender woman
9	16	White British	Sixth form or college student	Gay	Cisgender man
10	19	White British	Sixth form or college student	Gay	Transgender man
11	20	Asian Vietnamese	University student	Bisexual	Cisgender woman
12	16	White	Sixth form or college student	Queer	Questioning
13	19	White	University student	Asexual	Cisgender woman
14	20	Mixed (White and Asian)	University student	Lesbian	Cisgender woman
15	22	White	Full-time employment	Lesbian	Cisgender woman
16	18	White British	Sixth form or college student	Pansexual	Cisgender woman

A summary of the baseline assessments (*M*; SD) can be found in Table 3. Despite relatively high suicide risk (*M* = 11.94; SD = 2.41), only one safeguarding procedure was triggered during the 7-ESM assessment. Following the participant’s wellbeing check and risk assessment, further escalation was not needed. During the 7-ESM assessment, five participants self-harmed. Two participants did not indicate why they had self-harmed, however 2 indicated that self-harm had occurred following difficult interactions with others and for one participant this was related to negative self-thoughts. Across participants all self-harm behaviours included in the ISASi were endorsed, with the most endorsed behaviours being cutting, pinching, and interfering with wound healing. At baseline, participants suggested the intrapersonal functions (e.g., sensation-seeking, affect regulation) (*M* = 1.20, SD = 0.40) were more relevant to their self-harm, than interpersonal functions (e.g., interpersonal influence or boundaries; *M* = 0.40; SD = 0.25).

**TABLE 3:** Baseline measures descriptives [mean (*M*); standard deviation (SD)].

	Total score	
	*M*	SD
**MSPSS**	4.99	0.99
*Significant other*	5.47	1.35
*Family*	4.20	1.45
*Friends*	5.30	1.27
**ISASii**	0.65	0.23
*Intrapersonal functions*	1.20	0.40
*Interpersonal functions*	0.40	0.25
**SBQ-R**	11.94	2.41
**HADS**		
*Anxiety*	13.56	4.23
*Depression*	9.25	3.15
**Adapted Internalised Homophobia scale**	2.26	0.86
**Outness Inventory**	2.77	1.10
**Transgender Identity**	4.65	1.67
*Pride*	3.22	1.92
*Passing*	5.14	1.90
*Alienation*	4.00	1.72
*Shame*	4.34	2.03
**GCLS**		
Cluster 1: Gender congruence	3.04	1.46
Cluster 2: Gender-related mental well-being and general life satisfaction	2.93	0.70

Due to missing data, analyses across the whole GCLS scale was not possible.

### Feasibility

#### Recruitment and retention

Across the 2.5-month recruitment period, 29 individuals responded to the study call; 75% of whom were through MQ Participate. From the 29 respondents, 16 provided valid consent forms, therefore the enrolment rate was 55.2%. Seven people did not respond following the initial email contact and follow-up emails. Two chose not to take part as they were too busy, one person was not currently experiencing self-harmful thoughts or behaviours, and one declined as they felt the compensation was not enough for the study. Two people were excluded as they did not meet the inclusion criteria (over 25-years-old, invalid GP details).

Of the final sample, all the participants completed both the baseline assessment and 7-day ESM assessment. Therefore, throughout the experimental phases of this study, the retention rate was 100%. Twelve participants (75%) agreed to take part in the post-ESM interview. Reasons for not taking part in the interview were not being able to fit the interview around medical appointments, multiple instances of forgetting to attend, and not returning the completed consent form despite reminders.

#### App feasibility

Over the 7-day ESM assessment period, 14 participants were able to log into the mEMA app at least once a day. Two of the participants missed all surveys for the final day of the study, while one logged in multiple times on the last day but did not complete the full survey each time. Neither participant flagged why they did not respond on the final day within the post-ESM interview. Despite this, participants generally reported that the 7 days was an appropriate test period within the post-ESM interviews.

From observation of the data, for eight participants the first question of social experiences and context would stop following their responses to whether they were with others physically or online. If they responded online, the following branching questions were not presented. This indicated that there was a logic break between the design platform and the app. The remaining participants did not encounter this break. Potentially, this is a barrier to usability based on phone type. Phone type was not recorded in this study. However, this limitation was mentioned by a participant who owned a Microsoft phone, and previous studies have found technical issues of the mEMA app relating to phone type (29).

#### Adherence

Adherence to the ESM protocol was operationalised in three ways; (i) total responses to surveys and descriptives of response patterns; (ii) adherence to ESM topic surveys; (iii) participant adherence. Firstly, total number of responses to surveys was examined. For each participant, 42 surveys were sent over the course of the 7-day assessment period, resulting in 672 possible surveys to complete across the whole sample. The total number of responses to these surveys was 454 (67.6%). The highest response rates were on day 2 (77%), while the lowest responses were on days 4 (59.4%) and 7 (57.3%). On average, participants completed 4.05 (SD: 1.06) surveys per day.

Secondly, adherence was examined in relation to ESM topic surveys. This breaks down the ESM survey into specific topic items (social context, mental health, identity and minority stressors, and self-harm). Participants were asked about self-harm thoughts, with and without suicidal intention, and self-harm behaviour seven times. On average participants responded to 70.6% of these surveys (*M*: 4.94; SD: 1.24). All other ESM topic surveys were asked 42 times as they occurred in each survey. Similar adherence rates were seen across social context (63.1%; *M*: 26.3; SD: 6.5), mental health difficulties (65.0%; *M*: 27.3; SD: 7.4), and identity and minority stressor items (65.5%; *M*; 27.5; SD: 7.5).

Thirdly, participant adherence to the ESM protocol was demonstrated if the LGBTQ+ young person completed all six surveys each day. Therefore, participant adherence was assessed by considering study adherence and adherence to ESM item group; see in Table 4. Participant adherence ranged from 13 to 40 survey responses. The highest rate of completion was 95.2%, with another four participants being able to respond to over 80% of the total surveys. The lowest overall adherence was by two participants, who responded to less than 43% of the survey prompts.

**TABLE 4:** Participant adherence by total **experience sampling method** (ESM) survey adherence and ESM item group adherence; range, percentage, mean, and standard deviations.

P#	Range of survey responses per day	Total survey adherence completed *N* (%)	Average number of surveys responded to per day *M* (SD)	Completed *self-harm* items in surveys *N* (%)	Completed *social context* items in surveys *N* (%)	Completed *mental health* items in surveys *N* (%)	Completed *identity and minority stressor* items in surveys *N* (%)
P1	5–6	40 (95.2)	5.7 (0.5)	6 (85.7)	34 (81.0)	39 (92.9)	40 (95.2)
P2	2–6	33 (78.6)	4.7 (1.6)	5 (71.4)	33 (78.6)	33 (78.6)	33 (78.6)
P3	0–6	25 (59.5)	3.6 (2.1)	4 (57.1)	25 (59.5)	25 (59.5)	25 (59.5)
P4	2–6	33 (78.6)	4.7 (1.7)	4 (57.1)	29 (69.0)	31 (73.8)	31 (73.8)
P5	2–5	26 (61.9)	3.7 (1.1)	6 (85.7)	26 (61.9)	26 (61.9)	26 (61.9)
P6	4–6	35 (83.3)	5.0 (0.8)	5 (71.4)	33 (78.6)	35 (83.3)	35 (83.3)
P7	2–4	21 (50.0)	3.0 (0.6)	3 (42.9)	21 (50.0)	21 (50.0)	21 (50.0)
P8	5–6	36 (85.7)	5.1 (0.4)	7 (100.0)	34 (81.0)	34 (81.0)	34 (81.0)
P9	1–5	18 (42.9)	2.6 (1.3)	4 (57.1)	18 (42.9)	18 (42.9)	18 (42.9)
P10	2–5	24 (57.1)	3.4 (1.1)	6 (85.7)	23 (54.8)	23 (54.8)	23 (54.8)
P11	0–3	13 (31.0)	1.9 (1.1)	3 (42.9)	13 (31.0)	13 (31.0)	13 (31.0)
P12	2–5	25 (59.5)	3.6 (1.0)	6 (85.7)	25 (59.5)	25 (59.5)	25 (59.5)
P13	3–6	34 (81.0)	4.9 (0.9)	6 (85.7)	34 (81.0)	34 (81.0)	34 (81.0)
P14	2–5	23 (54.8)	3.3 (1.1)	4 (57.1)	22 (52.4)	21 (50.0)	21 (50.0)
P15	4–6	38 (90.5)	5.4 (1.0)	6 (85.7)	30 (71.4)	36 (85.7)	37 (88.1)
P16	3–6	30 (71.4)	4.3 (1.4)	4 (57.1)	20 (47.6)	23 (54.8)	25 (59.5)

### Acceptability

To determine the acceptability of the ESM study, LGBTQ+ young people were invited to take part in a post-ESM semi-structured interview. This would explore their perceptions of the ESM study, with a focus for the specific challenges and facilitators to taking part in this type of research, and opinions of how they felt the study could be improved. A total of four themes were developed, each containing subthemes. The thematic framework can be seen in Table 5. Themes and subthemes identified are detailed below with example quotes.

**TABLE 5:** Thematic framework of barriers and facilitators of taking part in the experience sampling method (ESM) study.

Theme	Descriptors	Subtheme	Descriptor
**Self-reflection and awareness**	Participants tracking their own mood, reflecting on this and increased awareness of their personal influencers. This helped them to engage with the study.	**Improved understanding of mood** (facilitator)	Majority of participants found that the ESM study helped them to track and reflect on their mood. Specifically, this aided awareness of influences to their self-perceptions of LGBTQ+ identity.
		**“But with awareness kind of comes some intense lows and intense highs”** (facilitator/barrier)	As awareness grew, participants were more aware of their self-harm. Mainly participants didn’t feel there was a change in the frequency of these thoughts, and some actually used the study as a barrier to self-harm. However, one participant found that this triggered more self-harmful thoughts.
		**Future uses** (suggestion)	Potential therapeutic uses for mood tracking and integration with clinical services.
**Practicalities of the ESM surveys**	Participants opinions on the survey and app were mainly positive. However some experienced notification errors.	**Quick, easy, and minimal impact** (facilitator)	Participants did not feel as those taking part in the ESM study had a large impact to their day because it was so quick.
		**Notification system error** (barrier)	Some participants faced notification errors. Either notifications failed to present, or the notification would not be dismissed once the survey had been completed.
**Daily timeframe**	Participants thoughts on the ESM assessment timeframes (8:00-22:00).	**Missing morning notifications** (barrier)	Several participants missed morning notification due to sleeping patterns.
		**“negative thoughts more come at night”** (barrier)	Participants felt that 10pm was too early to capture their self-harm behaviour
		**Personalised timeframe** (suggestion)	Participants wanted to adjust the timeframes to better suit their lifestyles. It was suggested this would be beneficial during work or education hours.
**Suggestions for a future study**	Participants reflected on the relevance of questions and how to improve the study.	**Streamlining ESM items** (suggestion)	Participants offered two suggestions to improve ESM surveys. These changes were related to the ESM items. These suggestions were separating cisgender and gender diverse items, and including additional self-harm items.
		**System changes and additional context** (suggestion)	Participants suggested a system which would allow for their experiences to be captured if they missed several surveys. They also wanted an option to write context for themselves or others to understand why their mood, thoughts or behaviours had changed.

#### Self-reflection and awareness

A key facilitator to engagement was the ability for participants to track their mood over time. This resulted in participants feeling that they had an increased awareness of their experiences, mood, thoughts, and feelings about self-harm. This allowed participants to reflect on their triggers and influences on their mood. Many participants found that this was helpful for them. Participants also suggested that aspects of ESM could be used in therapeutic or clinical services.

##### Improved understanding of mood

Most participants found that that the ESM study helped them to track and reflect on their mood. This was beneficial to their own wellbeing, as well as, helping them to engage with the study; *“It might have affected my mood for the better really because being able to check in and reflect is, was helpful for me.” (P10, gay, transgender man).* This enhanced understanding dominated most of the interviews. Some participants even made efforts to change their behaviours when noticing that they were scoring highly for depression or anxiety.

*“And I think, I don’t know, it was kind of like someone just checking in and being like “hello! You okay?” and being able to be like “actually no I’m not” like you know it was very useful to motivate me to be like right let’s change my mood, let’s improve how I’m feeling because that reflection wasn’t you know, I feel like shit a bit. [laugh]” (P2, gay, cisgender man).*

From this improved understanding of their mood, a number of participants became aware of how experiences which related to their LGBTQ+ identity could influence their mood and thoughts; *“Actually helped me understand a lot about myself, and how, how actually that could be effecting my mental health. Because I realised for some of the questions that I’ve been answering, they reflected on, that it actually, there was some correlation to it.” (P3, pansexual, non-binary).*

The ability to self-reflect widely encouraged participants to engage with the ESM study. By completing surveys, they were able to obtain a better reflection of their wellbeing and make their own evaluations of what influenced their mood and self-harm.

##### “But with awareness kind of comes some intense lows and intense highs”

As self-awareness and reflection grew, participants also commented how they were more aware of their self-harmful thoughts and behaviours. For most this caused no impact. Participants did not feel that they experienced more frequent or intensive self-harm than usual despite being asked daily; *“Erm, no I don’t think so. It didn’t make them worse or better [thoughts], in a way it was the same.” (P6, bisexual, cisgender woman).* Some found that they were able to use their engagement with the study as a barrier to self-harm behaviour. One participant mentioned how they were able to reflect on whether acting on their self-harmful thoughts was necessary, while another specified that she actively did not self-harm due to being in the study.

*“I feel like it made me more aware of them [thoughts], especially when it came to erm like self-harm [behaviour]. When I would be looking back on it, I’d be like well “I have thought about it but have I actually*…*? But I didn’t do it and now looking at it did I need to?” (P3, pansexual, non-binary)*.

However, with greater self-awareness of self-harm, a few participants did mention that they could, in certain circumstances, see that responding to questions about self-harm daily could be difficult. One participant discussed that if they were having a bad week (frequent self-harm ideation) they would have been less likely to engage with the study, while another disclosed they had more impulses to self-harm during the study. However, their greater self-awareness also acted as a barrier to engaging with this self-harm.

*“So I started to overanalyse my, essentially my emotions and everything [*…*] Yeah well it was triggering in that I felt like I had a bit of an impulse to do like, you know, bad things [self-harm]. But I say I managed to control it, because I was more well aware of how I was feeling and I knew what to do.” (P4, bisexual, cisgender woman)*.

##### Future uses

Several participants mentioned that they found the ESM study so useful to track mood and their self-harm that they felt aspects of experience sampling could be used within therapeutic or clinical services. The benefit of this would be that instead of being asked about their thoughts and feelings over the last 2 weeks, clinicians would be able to see within- and between- day changes. One participant, who was a medical student, discussed how the questions regarding mood and self-harm could be useful within in-patient settings or in the community to gain real-time reflections of risk.

*“I think that would be really useful, definitely in an in-patient setting and maybe even like if someone you feel is in a community setting and they’re really at risk, then getting them to answer these questions once a day, or 3 times or even 6 times a day, just to sort of check in and see what their risk is instead of waiting until someone is at crisis, and then saying “oh well we can’t help you now because you’re too ill” or whatever.” (P1, bisexual/demisexual, cisgender woman)*.

#### Practicalities of the experience sampling method surveys

The second theme presents the participants’ opinions of the overall survey and app itself. For most participants, aspects related to the ESM surveys facilitated their engagement with the study. This was primarily the speed and ease of completing ESM surveys. Due to these facilitators, participants felt that completing ESM surveys had very little impact to their daily lives. However, there was one element which acted as barrier for some participants: the notification system.

##### Quick, easy, and minimal impact

All participants mentioned that the ease of responding to the ESM surveys was a facilitator to their engagement with the study. A key aspect was that the surveys were short and therefore quick to complete, which had little impact to the participants’ activities; “… *because it’s just such a small snapshot and it takes so little time, you sort of do it and then you forget about it until you’ve got the next one to do, because it’s so quick that it doesn’t impact what you’re doing*…*” (P1, bisexual/demisexual, cisgender woman).*

Experience sampling method surveys were distributed through the mEMA app and accessed through personal phones; participants felt this made completing surveys easy. One participant reflected on how using an app rather than email, meant that there was less burden on the participant to remember to engage with the study; “…*using a phone app is definitely a good way to collect the data rather than just having something be like “please remember to fill in this form and email it to me X times per day”, that’s, it’s a good method*…*” (P9, gay, cisgender man).*

Participants did not feel that completing the ESM surveys was invasive, and the surveys had little impact on their wellbeing; *“It was [pause] I don’t know, fine to do? [laugh] That sounds really weird like, but it wasn’t stressful or felt overly invasive or anything.” (P15, lesbian, cisgender woman).* Due to the minimal impact of the study, it was encouraging that many participants mentioned how they would be happy to engage in other ESM studies.

##### Notification system error

A small number of participants experienced errors with the mEMA app’s notification system. For some this was that the app failed to present survey notifications. This meant that the participant had to actively go onto the app, find their survey schedule for the day and make their own alert system; “…*so it wouldn’t actually send me the notifications. So when I woke up I would literally have to check what the times were and set an alarm for each of them.” (P12, queer, questioning).*

However, for others if they had completed the survey, the two additional notification reminders would continue. This was mentioned as annoying; *“The thing is because it keeps notifying me even when I’ve done it, like buzz. And I’m like I’ve already done! Buzz, I’ve already done it! [laugh] To the app!” (P10, gay, transgender man).* Another participant found that the notification not automatically being dismissed meant that he wasn’t sure whether the current notification was new or a previous survey. This led to him missing survey notifications as he ignored further notifications.

“…*the technical problem I told you about where it wouldn’t automatically clear the notification after the window has expired. I remember, especially because it didn’t clear automatically, I had to manually do that so I only ever got the erm, self-harm end of the day survey I think twice*…*” (P9, gay, cisgender man).*

These notification errors, combined with the observational data which indicated a survey logic break for some participants (no branching questions), highlight a key barrier within this study. Aspects of the mEMA app appear to be unsuitable for study use.

#### Daily timeframe

The third theme concerns the primary barrier to engagement. This was the daily timeframe of 8:00–22:00 during which all ESM surveys were sent. This was related to most of the participants taking part during their summer holidays, as often they did not have specific daily schedules and therefore, they had variable sleeping patterns. Many felt that the surveys would start too early in the morning and end too early in the evening. It was suggested that participants had a personalised timeframe in future studies.

##### Missing morning notifications

Several participants highlighted within their interviews that they struggled to complete the surveys in the morning. This was related to participants waking up later on days when they did not have any scheduled plans such as work; *“I mean it was alright on the days I was in work because I get up early then but on the days I don’t I missed them, because like I woke up at like 2. [laugh]” (P12, queer, questioning).*

“…*I mean it was a bit hard to get all 6 erm, all 6 of the questionnaires in each day. Especially since my sleep schedule is absolute carnage, so I’ll often sleep in until about 11 and see I’ve missed a erm, [pause] I’ve missed my morning surveys*…*” (P9, gay, cisgender man).*

This acted as a barrier as 1-3 of the surveys could be presented before the participants were awake. Therefore, the number of responses was greatly reduced simply by the young person missing their notifications by being asleep.

##### “Negative thoughts more come at night”

A further barrier of the timeframe was that participants felt that 22:00 was too early to capture their self-harm behaviour; “…*with me I go to bed fairly late so by the time it asked that [self-harm] if something happened it wouldn’t have reflected anything.” (P6, bisexual, cisgender woman).* This indicates self-harm may not be captured by the final survey of the day which was distributed randomly between 20:00 and 22:00 each day.

“…*so a lot of these intrusive thoughts aren’t really into my head at that moment. It tends to come at night, so I feel if you had asked me during the nighttime, although I know that’s not a normal procedure to ask during the night, but I felt like it would have triggered more of a response from me [filling in surveys].” (P4, bisexual, cisgender woman).*

Therefore, this study may not have captured all self-harm, as participants may have gone on to engage with these behaviours but not recorded this in the next day’s survey. This builds into the specifications of how participants categorise their day, either midnight to midnight or their waking to sleeping period.

##### Personalised timeframe

To combat timeframe barriers, participants suggested having a personalised timeframe; “…*I think if there was more of a flexibility [*…*] if you could choose which hours you’d be more likely to fill stuff in from.” (P9, gay, cisgender man).* This would be adjusted around participants’ lifestyles; “…*the 8am all the way through maybe having it so many someone could put in their own timings, so say they have their own wake up and sleep. Say if they work night shifts then being able to adjust it for their own erm cycle.” (P3, pansexual, non-binary).*

One participant suggested that instead of just having a start and end time for each day, being able to block out specific time periods would be helpful when he was in college; *“That sort of thing, like having a timescale when it can asked but outside of that timescale don’t ask because I’m busy.” (P10, gay, transgender man).* Given the population of this ESM survey, this is an interesting suggestion for future studies to work around school, college, or university hours.

#### Suggestions for a future study

The final theme presents the participants’ reflections on the relevance of ESM questions and their perceptions of how to improve the survey for future studies. These suggestions were related to tailoring the ESM survey for gender identity, a further line of questioning regarding self-harm, and a procedure in place for participants who miss survey notifications or wish to offer further context for their own mood and self-harm.

##### Streamlining experience sampling method items

Some participants discussed changes to the ESM items. These changes focused on; (i) separating cisgender and gender diverse ESM questions; and (ii) including in-depth self-harm questions. Firstly a number of cisgender participants discussed how ESM items relating to misgendering and gender dysphoria were less relevant to them; *“I’d say the only thing that wasn’t useful was asking about gender dysphoria. [*…*] slightly tailor the questions to the individual. So if someone doesn’t have gender dysphoria don’t include those questions*…*” (P1, bisexual/demisexual, cisgender woman).* Some participants felt that removing these questions would save them time as they responded to each set of these questions the same. It was suggested that if at baseline assessment, someone stated that they are cisgender, they would not be presented with these questions.

However, a small number of cisgender participants found that these questions might be useful to capture any fluctuations in how they felt about their gender identity; “…*I feel like when gender dysphoria yeah sometimes I would answer like second to least one yeah, because like I’m not really struggling with it but I’d be like oh I’d have thoughts about it*…*” (P7, bisexual, cisgender woman).* It was suggested that tailoring ESM surveys to recognise gender identity more closely would be useful. However, dismissing these items by someone identifying as cisgender would miss some nuances of gender identity.

Secondly, several participants suggested changes to ESM items concerning self-harm. Given the precautions around self-harm items and the consideration of how frequently these were presented, participants mentioned that having more in-depth self-harm items would have benefits. One suggestion was to consider impulsivity, as this was associated with self-harm among some participants; “…*.it might have been quite helpful to ask about compulsive behaviours, if there were any compulsive behaviours or any impulsive decisions or something like that*…*” (P6, bisexual, cisgender woman).* This was recognised by participants as influential for moving from ideation to behaviour.

Another suggestion was distinguishing between someone actively self-harming and passively being injured. This was considered as a form of self-harm but potentially less directive or intentional. One of the participants who had endorsed self-harm within the 7-day ESM assessment mentioned that they were more likely to passively hurt themselves than actively self-harm.

“…*there was an option for have you deliberately hurt yourself. But there wasn’t an option for have you deliberately not got out of the way of harm. Which is like, not protecting yourself but not quite hurting yourself sort of thing, which I feel like might apply to people more. Because I know like if I’m frustrated or upset with myself, I’m less likely to go out of the way to protect myself from something bad happening.” (P10, gay, transgender man).*

Finally, one participant suggested that an ESM item considering the severity of self-harm should be included. This was suggested to distinguish between self-harm behaviours which might trigger the safeguarding procedure, rather than considering self-harm behaviour in conjunction with suicidal intention scores. This participant reasoned that by including this topic, researchers would be notified if someone had severely injured themselves, despite having low suicidal ideation.

*“So when it comes to questions like that like it needs to be more a severity thing because when it comes to it, I mean, like for instance snapping a band that is a form of self-harm. Well cut for me, cutting my leg. [*…*] Because I mean sometimes we get stuck in our own head that we don’t actually realise how badly we numb ourselves out and then cut and then it’s like oh that’s a bit deeper than I wanted it.” (P3, pansexual, non-binary).*

##### System changes and additional context

The final suggestion was including a system which would allow participants to report their experiences, mood, thoughts and feelings if they missed several surveys in one day. This would act as a reference for a chunk of time so that they had some data for the day; “…*like maybe if you miss a couple [surveys] it would be good to be like “hey this one [survey] is kind of going to be open until you do it” to kind of compensate for the ones you’ve missed maybe.” (P2, gay, cisgender man).* While this would not offer the same specific real-time data, it may aid engagement with the study. However, this could also cause participants to be less motivated to respond to each survey as they knew there was a back-up system in place.

Similarly, some participants discussed having a system in which they could provide context for their overall day. They indicated that this would be helpful for their own self-reflection to understand what had happened to cause low mood or self-harmful thoughts and behaviours that day, which could also be useful for research. This system could also potentially capture experiences which were influential outside of the ESM items asked.

*“Being able to put a little comment box at the end, oh I had a really bad argument. Just for myself looking back or anyone who wanted to look at it. It’s got some context for why I suddenly went like dipped really badly*…*” (P10, gay, transgender man).*

### Pilot data

To establish the sample size needed for a follow-up study, a power calculation was run (51). This indicated that between 190 and 210 participants would be required to obtain a strong sample power to determine effect sizes of 0.3, alpha of 0.05. This is based on an assessment period of 7 days, in which participants are sampled six times a day, with the ability to conduct multilevel regression modeling between all ESM items.

Given the high levels of anxiety and depression at baseline, the relevant ESM items (GAD-7 and PHQ-9) are presented as examples of within- and between- person fluctuations over the 7-day ESM assessment (Supplementary material 2). Higher scores PHQ-9 (range: 9–63) and GAD-7 (range: 7–49) were associated with greater severity of depressive and anxiety symptoms. From observation of the total scores, anxiety and depression rates varied throughout the week. For example, within-participant three depressive and anxiety symptoms were rated very high on day 1 (PHQ-9 *M* = 48.17; GAD-7 *M* = 41.00) whereas on day 5 these were much lower (PHQ-9 *M* = 17.20; GAD-7 *M* = 10.20). Between participants, on day 1 PHQ-9 ranged from an average of 15.33 to 48.17, while later in the week (day 6) these were slightly lower (13.50–47.50), despite two participants self-harming that day.

**FIGURE 2:**
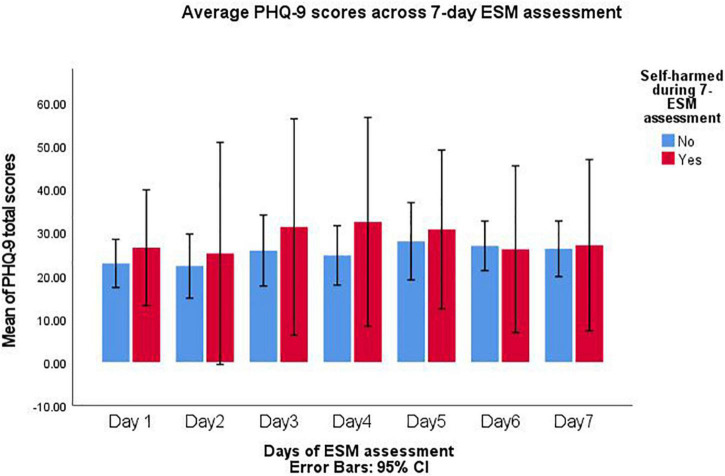
PHQ-9 scores compared between participant who self-harmed and those who did not during 7-day experience sampling method (ESM) assessment.

**FIGURE 3:**
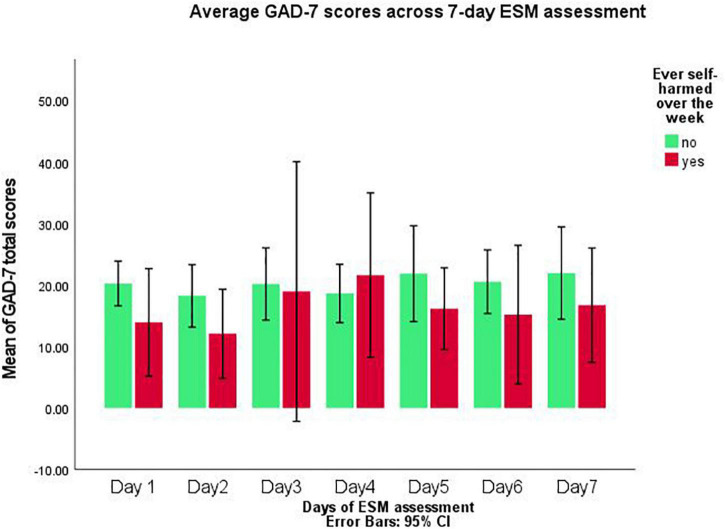
GAD-7 scores compared between participant who self-harmed and those who did not during 7-day experience sampling method (ESM) assessment.

Within Supplementary material 2, participants who self-harmed are highlighted on the days they acted on these behaviours. Considering the GAD-7 and PHQ-9 across participants who self-harmed within the study (*n* = 5) compared to those who did not (*n* = 11); depressive scores (PHQ-9) were consistently higher among participants who self-harmed (Figure 2). Whereas, anxiety (GAD-7) was typically higher among those who did not self-harm (Figure 3). This indicates that there are different relationships between underlying stressors and self-harm. Within a fully powered, follow-up study such associations could be considered to determine the temporal influence of stressors onto self-harm.

## Discussion

This study is the first to examine ESMs within LGBTQ+ young people who have current experiences of self-harm, with and without suicidal intentions. The overall findings support the feasibility and acceptability of ESM among this population. Several factors were mentioned which could improve the acceptability of the study for future research. From the pilot data, a larger sample size is needed for full complex analysis to establish temporal relationships between precipitating stressors and self-harm. This would be able to extend on the ESM pilot data, which demonstrated item scores varied within this study between- and within- participants, and offer evidence as to whether there were temporal influences of such precipitating stressors to self-harm in this population.

In relation to previous research, the enrolment rate for the study was comparable to other small feasibility studies (54, 55). Each of these studies included 14 participants (54, 55). However, compared to feasibility studies which considered high-risk adolescents and self-harm, enrolment is much lower (*n* = 34, (28); *n* = 53, (29)). Potentially these higher enrolment rates are related to the period of recruitment, as neither paper mentioned how long recruitment was open for these studies (28, 29), while this recruitment was limited to 2.5 months. The retention rate of participants, however, was consistent with previous ESM research in samples who have self-harm experiences (22, 26, 27). Indeed, retention of all participants was a particular strength of this study, on the higher end of retention rates comparably (56). Overall, adherence to survey completion (68%) was similar to other ESM studies considering adolescents and young people who experience self-harm [69%, (28); 63% (29). A barrier reported by participants were the daily timeframes (8:00–22:00), as often they were not awake for the first few assessments and felt that finishing assessments before 22:00 missed some potential behaviours. Future consideration should be given to personalised wake and sleep times, which could more accurately reflect a young person’s daily activities. A further barrier to study adherence were errors relating to the notification system. This was only experienced by a few participants, however, when considering in relation to the logic break this indicates that alternative platforms may be more efficient for ESM studies.

The long-term goal of this line of research inquiry is to understand how daily experiences prospectively influence self-harmful thoughts and behaviours among LGBTQ+ young people. This could then be used to inform future interventions or prevention strategies. ESM has been adapted to provide in-the-moment interventions to support other health behaviours (57, 58), through processes such as self-monitoring. One key theme of the ESM qualitative interviews highlighted the utility of ESM to enhance awareness and reflecting on their mood and self-harm. This was discussed as a therapeutic tool for themselves, acting as a barrier to their self-harm. Previous evidence has indicated the effectiveness of using ESM as an intervention (59). In their study, ESM provided personalised feedback, and was found to be as effective as a therapeutic tool among depressed individuals (59). Thus, it is possible ESM may have therapeutic application for those who self-harm as well. This would provide an individualised, easy to access, and relatively cheap way to reduce self-harm within LGBTQ+ young people.

Despite LGBTQ+ young people who self-harm being considered a high-risk population (7, 60, 61), there was only one event in which the safeguarding procedure was flagged. This event did not need to be escalated when speaking with the participant during their wellbeing check. The procedure followed a similar strategy to Glenn et al. (29), whereby participants would be contacted by the researcher within 24 h for a wellbeing check. This information is useful, firstly, to demonstrate that ESM with a high-risk population is possible. Secondly, it is ethical to conduct such research, as from the qualitative interviews’ participants found the ESM design highly helpful to monitor their self-harm and mood. Rather than feeling as though the survey assessments triggered their self-harm. Thirdly, to determine that this safeguarding procedure was acceptable to LGBTQ+ young people. All participants were told before taking part in the study that this safeguarding procedure would be in place to ensure participant safety; only one person did not give valid GP details and was therefore excluded. Considering this and previous research, it appears that ESM designs are appropriate to use with high-risk young people who experience self-harm (27–29, 62, 26).

### Strengths and limitations

This study demonstrates ESM is feasible, safe and acceptable with LGBTQ+ young people who experience self-harm. These findings are supported by the reflections of barriers and facilitators for study engagement. These demonstrate how to improve the study for participants and can be considered with development strategies in mind (e.g., research costs, ethical submissions and approvals).

The key limitation of this study is the recruitment period. Due to COVID-19, the start of this study was delayed. This followed in-depth team discussions and codesign with the LGBTQ+ Advisory Group. This meant there was only 2.5 months for recruitment to be conducted before the mEMA software license expired, resulting in a small sample. Furthermore, given that participants were only assessed 6 times a day over 7 days, expected missing data was not strongly accounted for. This needs to be considered for a follow-up study, as a minimum number of survey responses is needed to achieve statistical power and capture an effect. Therefore, if this study design was followed, a need for a much larger sample size (190–210 participants) is required.

## Conclusion

This study has indicated that it is feasible and acceptable to conduct ESM studies with LGBTQ+ young people with current self-harm experiences. There is worth in conducting a follow-up study with a greater number of participants, which would be able to determine the temporal relationships between precipitating stressors and self-harm. From this, we would be able to identify key moods, experiences or thoughts which might be targeted during self-harm interventions.

## Data availability statement

The original contributions presented in the study are included in the article/Supplementary material, further inquiries can be directed to the corresponding author/s.

## Ethics statement

The studies involving human participants were reviewed and approved by Science, Technology, Engineering and Mathematic Ethical Review Committee. Ethical approval: ERN_201745. Written informed consent from the participants’ legal guardian/next of kin was not required to participate in this study in accordance with the national legislation and the institutional requirements.

## Author contributions

AW, JA, ET, andMMconceptualised the study. AW devised the study protocol, completed an Open Science protocol, and was responsible for data collection, analysis and the initial write up. JA, ET, and MM reviewed the drafts of the manuscript. All authors contributed to the article and approved the submitted version.

## Footnotes

1
https://participate.mqmentalhealth.org

2
https://mema.ilumivu.com/
