# Supplementary material for: Feasibility and acceptability of experience sampling among LGBTQ+ young people with self-harmful thoughts and behaviours
Source: Front Psychiatry. 2022 Aug 17;13:916164. doi: 10.3389/fpsyt.2022.916164 (PMC9428709; doi:10.3389/fpsyt.2022.916164)
Supplement: Supplementary file 1 [file Data_Sheet_2.docx]

**Supplementary Materials 2**

**Daily changes of ESM items over the 7-day ESM assessment period**

| **P#** | **Self-harm behaviour**  **(Y/N)** | **Self-harm ideation** | **Suicidal ideation** | **Depression (PHQ-9) (M)** | **Anxiety (GAD-7) (M)** |
| --- | --- | --- | --- | --- | --- |
| **DAY 1** | | | | | |
| P1 |  |  |  | 22.60 | 17.60 |
| P2 | N | 2 | 1 | 20.50 | 20.83 |
| P3 | N | 1 | 1 | 48.17 | 41.00 |
| P4 |  |  |  | 23.40 | 19.20 |
| P5 |  |  |  | 28.00 | 17.50 |
| P6 | N | 3 | 1 | 22.00 | 13.67 |
| P7 | N | 2 | 2 | 36.67 | 25.33 |
| P8 | N | 3 | 2 | 17.50 | 28.00 |
| P9 |  |  |  | 23.00 | 8.00 |
| P10 | N | 2 | 2 | 21.67 | 12.67 |
| P11 | N | 2 | 1 | 22.50 | 20.50 |
| P12 | N | 5 | 4 | 39.00 | 21.25 |
| P13 | N | 1 | 1 | 13.40 | 14.00 |
| P14 |  |  |  | 15.33 | 15.33 |
| P15 | N | 3 | 1 | 27.67 | 24.00 |
| P16 |  |  |  | 42.33 | 28.33 |
| **DAY 2** | | | | | |
| P1 | N | 2 | 2 | 25.83 | 21.17 |
| P2 | N | 4 | 1 | 17.67 | 21.00 |
| P3 |  |  |  | 27.80 | 25.60 |
| P4 | N | 1 | 1 | 27.83 | 20.80 |
| P5 | N | 3 | 1 | 33.40 | 22.80 |
| P6 | N | 2 | 1 | 18.17 | 14.50 |
| P7 | N | 1 | 2 | 27.50 | 24.75 |
| P8 | N | 1 | 1 | 15.17 | 16.00 |
| P9 | N | 1 | 1 | 15.00 | 7.00 |
| P10 | N | 1 | 1 | 18.00 | 9.67 |
| P11 | N | 5 | 3 | 25.00 | 30.00 |
| P12 | N | 7 | 5 | 49.20 | 17.00 |
| P13 |  |  |  | 9.00 | 7.33 |
| P14 | N | 1 | 1 | 9.25 | 7.25 |
| P15 | N | 3 | 1 | 33.83 | 22.67 |
| P16 | N | 5 | 4 | 40.50 | 34.67 |
| **DAY 3** | | | | | |
| P1 | N | 3 | 3 | 27.00 | 22.80 |
| P2 |  |  |  | 15.67 | 17.00 |
| P3 |  |  |  | 33.33 | 24.67 |
| P4 | N | 1 | 1 | 26.67 | 31.67 |
| P5 | N | 4 | 1 | 37.00 | 23.33 |
| P6 |  |  |  | 19.60 | 11.40 |
| P7 |  |  |  | 35.50 | 27.00 |
| P8 | N | 1 | 1 | 15.25 | 13.00 |
| P9 |  |  |  | 16.50 | 7.00 |
| P10 | Y | 7 | 5 | 49.00 | 37.00 |
| P11 |  |  |  | 13.33 | 15.67 |
| P12 |  |  |  | 39.75 | 20.25 |
| P13 | N | 1 | 1 | 12.80 | 10.20 |
| P14 | N | 1 | 1 | 19.67 | 11.00 |
| P15 | N | 4 | 1 | 42.20 | 25.20 |
| P16 | N | 6 | 5 | 40.67 | 35.00 |
| **Day 4** | | | | | |
| P1 | N | 4 | 3 | 26.00 | 23.25 |
| P2 | N | 1 | 1 | 23.50 | 16.50 |
| P3 | N | 1 | 1 | 29.50 | 20.00 |
| P4 | N | 1 | 1 | 29.00 | 25.80 |
| P5 | N | 6 | 2 | 38.40 | 26.60 |
| P6 |  |  |  | 19.50 | 17.75 |
| P7 |  |  |  | 32.33 | 21.00 |
| P8 | N | 1 | 1 | 13.80 | 14.80 |
| P9 | N | 1 | 1 | 19.00 | 11.50 |
| P10 |  |  |  | 45.00 | 28.50 |
| P11 |  |  |  | 12.33 | 7.67 |
| P12 | Y | 7 | 7 | 46.00 | 28.50 |
| P13 | N | 1 | 1 | 17.40 | 10.60 |
| P14 | N | 1 | 1 | 11.50 | 9.75 |
| P15 |  |  |  | 29.75 | 19.00 |
| P16 |  |  |  | 54.50 | 32.00 |
| **DAY 5** | | | | | |
| P1 | N | 4 | 3 | 27.00 | 24.83 |
| P2 | N | 1 | 1 | 38.80 | 18.80 |
| P3 | N | 1 | 1 | 17.20 | 10.20 |
| P4 |  |  |  | 39.33 | 34.33 |
| P5 | N | 2 | 1 | 36.75 | 26.00 |
| P6 | N | 2 | 1 | 19.80 | 17.00 |
| P7 | N | 2 | 2 | 38.00 | 20.67 |
| P8 | N | 4 | 5 | 27.00 | 37.20 |
| P9 |  |  |  | 26.00 | 10.00 |
| P10 | N | 2 | 5 | 30.00 | 19.00 |
| P11 | N | 1 | 1 | 24.00 | 21.00 |
| P12 | N | 7 | 7 | 46.75 | 18.50 |
| P13 | N | 1 | 1 | 10.40 | 9.20 |
| P14 |  |  |  | 9.67 | 7.00 |
| P15 | N | 3 | 1 | 24.17 | 18.17 |
| P16 | N | 5 | 5 | 44.33 | 37.40 |
| **DAY 6** | | | | | |
| P1 | N | 3 | 3 | 28.33 | 24.67 |
| P2 | N | 1 | 1 | 17.50 | 11.17 |
| P3 | N | 1 | 1 | 13.50 | 7.00 |
| P4 | N | 5 | 2 | 26.67 | 29.80 |
| P5 | N | 3 | 2 | 36.75 | 30.00 |
| P6 | Y | 7 | 2 | 20.75 | 20.50 |
| P7 |  |  |  | 36.67 | 22.33 |
| P8 | N | 7 | 1 | 14.50 | 16.50 |
| P9 | Y | 1 | 1 | 24.67 | 9.33 |
| P10 | N | 2 | 3 | 15.50 | 8.75 |
| P11 |  |  |  | 19.50 | 14.50 |
| P12 | N | 7 | 7 | 43.33 | 22.00 |
| P13 | N | 1 | 1 | 28.20 | 15.00 |
| P14 | N | 4 | 2 | 24.50 | 15.00 |
| P15 | N | 3 | 1 | 28.50 | 20.00 |
| P16 | N | 6 | 5 | 47.50 | 34.50 |
| **DAY 7** | | | | | |
| P1 | N | 4 | 3 | 27.50 | 24.50 |
| P2 |  |  |  | 19.80 | 21.80 |
| P3 |  |  |  |  |  |
| P4 |  |  |  | 28.50 | 41.00 |
| P5 | N | 3 | 2 | 36.00 | 28.67 |
| P6 | N | 4 | 2 | 20.50 | 18.50 |
| P7 |  |  |  | 37.67 | 22.33 |
| P8 | N | 1 | 1 | 12.20 | 9.80 |
| P9 | N | 2 | 3 | 28.00 | 13.50 |
| P10 | N | 1 | 1 | 15.50 | 10.75 |
| P11 |  |  |  |  |  |
| P12 | N | 7 | 7 | 44.00 | 24.00 |
| P13 | N | 1 | 1 | 24.00 | 9.80 |
| P14 |  |  |  | 18.50 | 15.00 |
| P15 | Y | 5 | 1 | 31.25 | 24.25 |
| P16 |  |  |  |  |  |
